# Analysis of miRNA expression profiles in exosomes of SMB-S15 cells treated with resveratrol

**DOI:** 10.1007/s00705-023-05884-6

**Published:** 2023-10-08

**Authors:** Qiang Shi, Lina Zhang, Xiemusiye Wulayin, Rundong Cao, Mingyuan Jiao, Jing Wang, Jun Han, Xiao-Ping Dong, Chen Gao

**Affiliations:** 1 Tongzhou Maternal and Child Health Hospital of Beijing, Beijing, 101100 China; 2 State Key Laboratory for Infectious Disease Prevention and Control, Collaborative Innovation Center for Diagnosis and Treatment of Infectious Diseases (Zhejiang University), Chinese Center for Disease Control and Prevention, National Institute for Viral Disease Control and Prevention, Chang-Bai Rd 155, Beijing, 102206 People’s Republic of China; 3 National Key Laboratory of Intelligent Tracking and Forecasting for Infectious Diseases Center, Chang-Bai Rd 155, Beijing, 102206 China

## Abstract

Exosomes are double-layered vesicle bodies secreted by cells, in which microRNAs (miRNAs) play an important role. In a previous study, we found that treatment of the prion-infected cell line SMB-S15 with resveratrol can effectively inhibit the propagation of PrP^Sc^ *in vitro* and eliminate its infectivity *in vivo*. In this study, the global expression profiles of miRNAs in extracellular exosomes during resveratrol clearance of PrP^Sc^ in SMB-S15 cells were analyzed. Extracted exosomal miRNAs from the prion-infected cell line SMB-S15 (S15) and its normal partner cell line SMB-PS (PS) as well as SMB-S15 cells exposed to resveratrol for 4 days (RES4) and 8 days (RES8) were subjected into deep sequencing. Similarities and differences in the levels of differentially expressed miRNAs as well as the signaling pathways that are potentially involved were comparatively analyzed. The possible influences on the expression of genes affected by changes in exosomal miRNAs in the context of the prion pathway were further analyzed. These alterations in exosomal miRNA levels may help us to understand the functional transmission of intercellular messages and the pathogenesis of prion biology and prion disease.

**Supplementary Information:**

The online version contains supplementary material available at 10.1007/s00705-023-05884-6.

## Introduction

Prion disease is a neurodegenerative disorder that affects humans and a variety of mammals, characterized by a long incubation period and a 100% fatality rate. Human prion diseases include kuru, Creutzfeldt-Jakob disease (CJD), fatal familial insomnia (FFI), and Gerstmann-Sträussler-Scheinker syndrome (GSS). Animal prion diseases include scrapie, chronic wasting disease (CWD), and bovine spongiform encephalopathy (BSE) [1]. The pathogenic mechanism of prion disease involves conformational changes in a normal cellular prion protein (PrP^C^) to its pathological isoform (PrP^Sc^), resulting in an increase in the β-sheet content and a decrease in the α-helix content in the protein secondary structure. Although the mechanism of the spread of prions between cells is not clear, it has been proposed to be mediated by intercellular contact, nanotubes, or exosomes [2–4].

Exosomes are double-layered vesicle bodies secreted by cells. They range in size from 30 to 150 nm and are produced from the cell membrane as a result of endocytic processes. Endocytosis forms endocytic bodies (endosomes). With the help of the protein ESCRT (endosomal sorting complex required for transport) and regulatory proteins, the endosome buds inward to form multiple luminal vesicles (intraluminal vesicles, ILVs). The endosome contains multiple luminal vesicles and becomes a multivesicular body (MVB), which merges with the cell membrane and is released as an exosome. Exosomes contain a large variety of proteins, lipids, DNA, RNA, and other substances and differ from intracellular vesicles [5]. Exosomes are likely to be involved in the development of neurodegenerative diseases, and it has been proposed that prions might be transmitted between cells through the exosomal pathway [5, 6].

MicroRNAs (miRNAs) are non-coding endogenous RNAs consisting of 18-24 ribonucleotides, and they are key components of exosomes. miRNAs can bind to the 3'-untranslated region of the target mRNAs, leading to their degradation and causing a reduction in the expression of the target protein [7]. miRNA thereby exerts a significant regulatory role in the process of gene transcription. miRNAs from one cell may enter another cell via the spread of exosomes. How the expression of miRNAs in exosomes changes during prion replication in host cells remains unclear.

Resveratrol is a polyphenolic compound that is mainly derived from plants such as peanut, grape, *Polygonum cuspidatum*, and mulberry. Resveratrol is an active molecule involved in scavenging free radicals and anti-cell-membrane lipid peroxidation. It is believed to be potentially useful for treatment of aging-related diseases, such as neurodegenerative disorders, cancer, and metabolic disorders [8]. In a previous study, we found that treatment of prion-infected SMB-S15 cells with resveratrol can effectively inhibit the propagation of PrP^Sc^ *in vitro* and eliminate its infectivity *in vivo* [9].

In this study, the global expression profiles of miRNAs in extracellular exosomes during resveratrol clearance of PrP^Sc^ in SMB-S15 cells were studied. The extracted exosomal miRNAs from the prion-infected cell line SMB-S15 (S15) and its normal partner cell line SMB-PS (PS), as well as SMB-S15 cells exposed to resveratrol for 4 days (RES4) and 8 days (RES8) were subjected to deep sequencing. The similarities and differences in the levels of differentially expressed miRNAs, as well as signaling pathways that are potentially involved, were investigated.

## Materials and methods

### Cell culture, exosome collection, and RNA extraction

The cell line SMB-S15, which is stably infected with the scrapie agent Chandler strain, and its normal partner cell line SMB-PS, obtained from the Roslin Institute, UK [10], were maintained in OptiMEM supplemented with 10% fetal calf serum in a humid atmosphere with 5% CO_2_ at 37°C. After growing to 75%, SMB-S15 cells were exposed to 10 μM resveratrol dissolved in dimethyl sulfoxide (DMSO) for 4 and 8 days, and these cells were designated as “SMB-RES4” and “SMB-RES8”, respectively. In addition, SMB-S15 and SMB-PS cells were incubated with the same amount of DMSO without resveratrol as controls. All four of these cell preparations were cultured with serum-free medium for another 48 hours after withdrawal of resveratrol (MCE HY-16561, purity>99.7%) or DMSO, after which the supernatants were collected for extraction of exosomes.

The collected supernatants from each cell preparation were centrifuged for 15 min at 3,000 *g* in order to remove cellular debris. The exosomes were extracted using an ExoQuick-Tc kit (SBI). Briefly, 5 ml of each supernatant was mixed with 1 ml of ExoQuick-Tc and kept at 4°C overnight. The solutions were centrifuged at 13,000 rpm for 2 min, and the pellets were mixed with the lysis buffer provided in the kit and vortexed for 15 s. The miRNAs in the collected exosomes were extracted using a SeraMir Exosome RNA Purification Kit (SBI) according to the manufacturer’s instructions. The RNA concentration in each sample was determined photometrically at 260 nm using a NanoDrop spectrophotometer (PeqLab), and 5 μg of each sample was used for ultra-deep sequencing.

### Western blot

The proteins in exosome samples were separated by 15% SDS-PAGE and electroblotted onto a nitrocellulose membrane using a semi-dry blotting system (Bio-Rad). Membranes were blocked at room temperature (RT) for 1 h with 5 % (w/v) nonfat milk powder in 1× Tris-buffered saline containing 0.1% Tween 20 (TBST) and then probed at 4°C overnight with primary antibodies, including anti-PrP antibody (6D11, SantaCruz, sc-58581), anti-β-actin antibody (Subrray Biotechnology, sr-25113), ALIX antibody (Abcam, ab275377), TSG101 antibody (Abcam, ab125011), and calnexin antibody (Abcam, ab22595). After washing with TBST, the blots were incubated with horseradish peroxidase (HRP)-conjugated goat anti-mouse antibody (Jackson ImmunoResearch Labs, 115-035-003 and 111-035-003) at RT for 2 h. The blots were developed using an enhanced chemiluminescence system (ECL, PerkinElmer, NEL103E001EA) and visualized on autoradiography films (General Electric). Images were captured using a ChemiDoc™ XRS+ Imager (Bio-Rad). To detect the presence of proteinase K (PK)-resistant PrP^Sc^, the exosome samples were digested with PK at a final concentration of 25 μg/ml at 37°C for 60 min prior to Western blotting. The PK digestion was terminated by heating the samples with loading buffer at 100°C for 10 min.

### Deep sequencing of small RNAs and bioinformatics analysis

Construction of cDNA libraries, cluster generation, and subsequent ultra-deep sequencing on a Solexa/Illumina platform were performed at Beijing Genomics Institute Tech (BGI), Shenzhen, China. In brief, 5 μg of total RNA from each sample was size-fractionated by 15% polyacrylamide gel electrophoresis (PAGE). The small-RNA fraction (18-30 nt) was extracted and ligated to 5’ and 3’ RNA adaptors using T4 RNA ligase. The RNA-adaptor constructs were then purified and reverse transcribed. The reverse-transcribed products were amplified using the following polymerase chain reaction (PCR) program: 98°C for 30 s, followed by 15 cycles of 98°C for 10 s, 72°C for 15 s, and 72°C for 10 min. Ultra-deep sequencing was performed using one flow cell channel per sample.

The data analysis process routinely used at BGI is summarized below. First, the sequence tags from ultra-deep sequencing underwent data cleaning to remove low-quality tags and 5' adaptor contaminants. Next, the software AASRA [10] (https://github.com/biogramming/AASRA) was used to compare the clean reads to the reference and other small-RNA databases, except that Rfam was aligned using cmsearch [11]. In addition, the length distribution of the clean tags with common and specific sequences between samples was summarized. The comparison and annotation of all sRNAs with various RNAs was also summarized. In the above annotation information, there may have been cases in which the same sRNA was simultaneously compared to two different sources of annotation information. In an effort to obtain a unique annotation for each unique sRNA, the sRNA annotation was performed in the following order of priority: miRNA > piRNA > snoRNA > Rfam > other sRNA. The identified known miRNAs were subjected to analysis for differential expression, clustering, and KEGG pathway identification.

The transcripts per million (TPM), i.e., for each 1,000,000 RNA molecules in the RNA-seq sample, was determined to normalize the expression levels of small RNAs and thereby lessen the impact of varying numbers of sequence reads on quantitative accuracy. The standardized data were then used directly for subsequent differential comparison analysis, and normalized data were used to calculate the fold change and *P*-value. If an miRNA had no reads, the normalized read count for this miRNA was set at 0.01. miRNAs with more than a twofold change (shown in log 2.0) and a *P*-value less than 0.01 compared to the normal control were considered differentially expressed miRNAs.

### Quantitative real-time PCR (qRT-PCR)

To verify the expression status of the miRNAs, quantitative real-time PCR (qRT-PCR) assays were performed using an All-in-OnemiRNA qRT-PCR Reagent Kit (GeneCopoeia, USA). The specific primers for mmu-miR-182-5p, mmu-miR-142-3p, mmu-miR-532-5p, mmu-miR-7b-5p, mmu-miR-100-5p, mmu-miR-243p-1, mmu-miR-31-5p, and mmu-miR-122-5p were synthesized by GeneCopoeia, China. The PCR reaction mixture contained 2 μl of first-strand cDNA, 10 μl of 2× all-in-one qmix, 2 μl of all-in-one primer, 2 μl of universal adapter primer, 0.4 μl of 50× ROX dye, and 3.6 μl of ddH_2_O in a final volume of 20 μl. After initial denaturation at 95°C for 10 min, thermal cycling was performed for 40 cycles at 95°C for 10 s and 60°C for 45 s in a 7500 Real Time PCR System (Applied Biosystems). The qRT-PCR procedure was repeated more than three times for each miRNA.

## Results

### Preparation of exosome extracts from different SMB cells

**Fig. 1:**
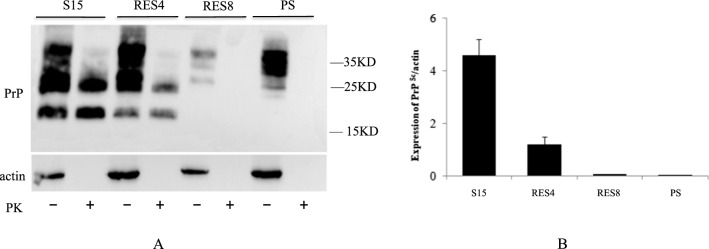
Western blots for PrP^Sc^ in SMB-S15, -PS, -RES4 and -RES8 cells. (A) Representative Western blot. PK-, without PK digestion; PK+, with PK digestion. The molecular weights are showed on the right. (B) Quantitative representation of the average gray values of PrP^Sc^ signals in various preparations normalized to the data for β-actin in the preparations without PK treatment. Each experiment was repeated at least three times. Graphical data are the mean ± SD. The Western blot assay was performed using total lysates prepared from cells in that were treated for 8 h with or without 10 nM resveratrol. PrP was detected using mAb 6D11. The concentration of PK was 25 μg/ml. The protein concentration was determined by BCA assay (Pierce), and 3050 μg of total protein was subjected to 12% SDS PAGE. The relative band intensity in ECL-developed Western blots was measured by densitometry, using ImageJ software.

To investigate potential changes in miRNA levels in exosomes during clearance of prions, the prion-infected cell line SMB-S15 was incubated with 10 μM resveratrol (dissolved in DMSO) and maintained for 4 (SMB-RES4) and 8 (SMB-RES8) days, respectively. In our previous research [11], SMB-S15 cells were exposed to different concentrations of resveratrol (from 0.25 to 200 μM) for 7 days, and PrP^Sc^ was found to be almost undetectable at resveratrol concentrations of 5.0 μM or more. Therefore, 10 µM was selected as the working concentration of resveratrol in this study. SMB-S15 and SMB-PS cells were also exposed to the same amount of DMSO without resveratrol for 8 days. Prior to extraction of exosomes, the presence of PrP^Res^ in the cells was evaluated by proteinase K (PK) treatment and Western blot assay. As shown in Fig. 1, PrP^Res^ signals were observed in the lysates of SMB-S15 cells, but not in its normal partner cell line SMB-PS. Markedly weaker PrP^Res^ signals were detectable in lysates of SMB-RES4 cells, but not in lysates of SMB-RES8 cells.

**Fig. 2:**
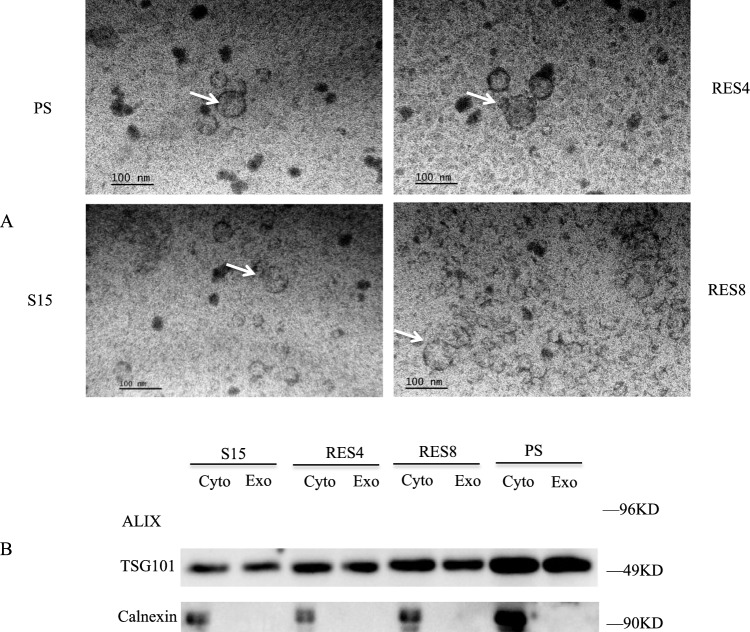
Analysis of extracted exosomes from four different SMB cell derivatives. (A) Representative electron microscopy images after negative staining, showing numerous vesicle-like structures with sizes varying from 50-120 nm, which were present in all four cell types. (B) Western blots for quality control of exosomes extracted from cells that were treated for 8 h with or without 10 nM resveratrol. The exosomes in 5 ml of cell culture medium were extracted and suspended in 40 µl of PBS. 10 µl of PBS was used as a loading control. This was found to correspond to 30-50 µg of protein (as determined using a BCA assay; Pierce) loaded onto each well of a 12% SDS PAGE gel. TSG101 and ALIX are biomarkers for exosomes. Calnexin is an ER protein that is not present in exosomes.

Exosomes from SMB-S15 cells with or without resveratrol treatment were extracted using a commercial kit. Morphological examination by electron microscopy after negative staining revealed numerous vesicle-like structures varying in size from 50 to 120 nm (Fig. 2A). No significant differences in the number or size of the vesicles were observed among the four preparations. The quality of the extracted exosomes was analyzed by Western blot using representative markers of exosomes, including tumor susceptibility gene 101 (TSG101) and apoptosis ALG-2 interacting protein X (ALIX), as well as the ER marker calnexin, which is not present in exosomes [12]. The Western blots showed that both TSG101 and ALG-2 were present in the exosome and cytoplasm fractions of all of the cells tested, whereas calnexin was detected only in fractions from the cytoplasm (Fig. 2B). This demonstrates that the preparation of extracted vesicles was mainly composed of exosomes.

### Up- and downregulation of miRNAs in the exosomes of different SMB cells

The extracted miRNAs from the exosomes were subjected to Solexa sequencing after the RNA quality control. The number of clean reads obtained by Solexa sequencing, excluding reads containing ambiguous bases and adaptor contaminants, was 22,533,939 (81.19%) for SMB-PS cells, 30,440,834 (79.59%) for SMB-RES4 cells, 24,524,557 (83.96%) for SMB-RES8 cells, and 23,464,220 (77.72%) for SMB-S15 cells. The majority of the reads in all cases were 18 to 24 nt in length, with a peak at 21 nt. After alignment to reference sequences from Rfam in the GenBank database, the sRNAs were classified into categories. In total, 782, 647, 830, and 848 known miRNAs were identified in SMB-PS, SMB-RES4, SMB-RES8, and SMB-S15 cells, respectively.

**Fig. 3:**
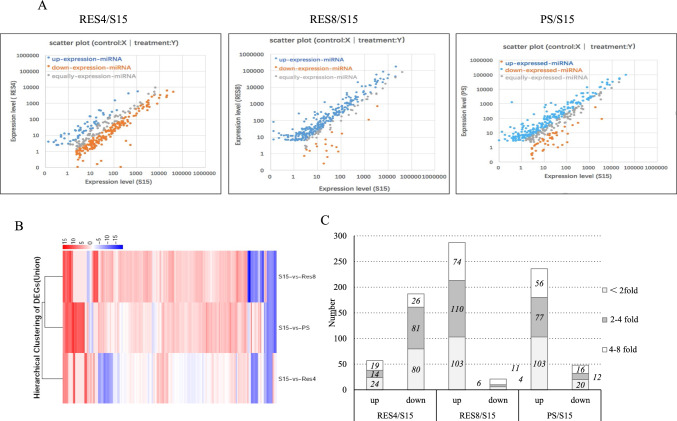
Comparison of differentially expressed exosomal miRNAs among the cell lines SMB-S15, -PS, -RES4, and -RES8. (A) Scatter plots of the differential expression of miRNAs in the groups RES4/S15, RES8/S15, and PS/S15. Red dots indicate upregulated miRNAs, blue dots show downregulated miRNAs, and grey dots show unchanged miRNAs. The expression levels (log2) of the samples (RES4, RES8, and PS cells) are indicated on the *y*-axis, and those of S15 cells are on the *x*-axis. B. Hierarchical clustering analysis of exosomal miRNAs that were differentially expressed among the cell lines. (C) Distributions of the upregulated and downregulated differentially expressed miRNAs in RES4, RES8, and S15 cells, respectively. The *y*-axis represents the number of miRNAs with altered expression in the different samples.

A comparison of exosomal miRNA levels in SMB-PS, SMB-RES4, and SMB-RES8 cells with those in SMB-S15 cells was used to identify differentially expressed miRNAs, as shown in Fig. 3A. Interestingly, the majority of the upregulated miRNAs (blue dots) were in SMB-RES8 and SMB-PS cells, in which there was no detectable PrP^Sc^, while downregulated miRNAs (orange dots) were predominantly found in SMB-RES4 cells, which contained smaller but repeatedly detectable amounts of PrP^Sc^ compared with SMB-S15 cells. All of the differentially expressed miRNAs identified in the exosomes of SMB-PS, SMB-RES4, and SMB-RES8 cells were subjected to hierarchical clustering analysis after being normalized to the value of each miRNA in SMB-S15 cells. As shown in Fig. 3B, the miRNA expression profile of SMB-RES8 cells was more similar to that of SMB-PS cells than to that of SMB-RES4 cells.

In comparison to SMB-S15 cells, the number of significantly upregulated and downregulated miRNAs was 57 (19 higher than 8-fold) and 187 (26 lower than 8-fold), respectively, in SMB-RES4 cells, 287 (74 higher than 8-fold) and 21 (11 lower than 8-fold), respectively, in SMB-RES8 cells, and 236 (56 higher than 8-fold) and 48 (16 lower than 8-fold), respectively, in SMB-PS cells (Fig. 3C). The most significantly up- and downregulated miRNAs in exosomes were miR-324-5p (9.05log2) and miR-7224-3p (-11.2log2) in SMB-RES4 cells, miR-3543 (12.56log2) and miR-7a-1-3p_1 (-17.20log2) in SMB-RES8 cells, and miR-3570 (13.96log2) and miR-132-5p (-12.69log2) in SMB-PS cells.

Among the differentially expressed miRNAs showing a change of 8-fold or more, 17 were observed in the exosomes of both SMB-PS and SMB-RES8 cells, including 10 that increased in abundance and seven that decreased (Table 1). Further analysis of the states of those 17 miRNAs in SMB-RES4 cells revealed a unique profile. Among the 10 upregulated miRNAs, six were decreased in SMB-RES4 cells, while among the seven downregulated miRNAs, five were increased in SMB-RES4 cells (Table 1). These data show that the profile of the exosomal miRNAs in SMB-RES8 cells is similar to that in SMB-PS cells, while that in SMB-RES4 is considerably different.

**Table 1:** Comparison of the status of miRNAs showing an 8-fold change in both SMB-PS and SMB-RES8 cells with that in SMB-RES4 cells

	log2 (PS/S15)	*P*-value	log2 (Res8/S15)	*P*-value	log2 (Res4/S15)	*P*-value
Upregulated						
mmu-miR-190b-5p	9.108337761	0	6.343661617	9.65E-64	4.754887502	1.9E-20
mmu-miR-24-3p_1	5.879396305	0	5.722376737	0	0.381015887	0.0000326
mmu-miR-3549	5.169082341	0	3.433200077	1.12E-137	-0.742753747	0.00785892
mmu-miR-99a-5p	5.105588289	0	3.270002265	0	-7.819519348	0
mmu-miR-122	5.001185939	2.97E-46	11.66753176	0	2.584962501	3.24E-08
mmu-miR-671-5p	4.692324899	2.15E-45	5.398743692	4.58E-106	-3.263034406	0.00695748
mmu-miR-194-5p	4.383943432	0	3.162001962	0	2.127862863	3.26E-155
mmu-miR-146a-5p	3.737874604	0	3.032682842	0	-1.407991694	1.32E-230
mmu-miR-151-3p	3.455631717	0	3.016976777	0	-0.970575388	2.14E-18
mmu-miR-219a-5p	3.099729571	1.24E-27	4.570345405	1.96E-140	-1.096215315	0.0274208
Downregulated						
mmu-miR-142-5p	-13.23182118	2.81E-30	-4.136002615	3.72E-113	3.896152997	0
mmu-miR-132-5p	-12.6926155	5.17E-21	-12.6926155	1.45E-37	-0.223482482	0.219792
mmu-miR-1a-3p	-11.63934071	2E-10	-11.63934071	1.83E-18	0.369087913	0.1167654
mmu-miR-7b	-5.925999419	8.5E-223	-10.36340473	0	-2.799890649	3.93E-259
mmu-miR-142-3p	-5.510747408	1.16E-74	-4.136002615	3.72E-113	3.640376657	0
mmu-miR-1b-5p	-4.828163384	8.64E-174	-5.571255081	0	0.713359855	1.93E-49
mmu-miR-133c	-3.842893629	4.07E-96	-5.462502273	1.23E-206	0.714917265	5.26E-32

### Confirmation of the status of eight differentially expressed miRNAs by qRT-PCR

To confirm the status of differentially expressed miRNAs identified by Solexa sequencing, four upregulated (mmu-miR-182-5p, mmu-miR-142-3p, mmu-miR-532-5p, and mmu-miR-7b-5p) and four downregulated miRNAs (mmu-miR-100-5p, mmu-miR-24-3p-1, mmu-miR-31-5p, and mmu-miR-122-5p), all of which were significantly altered in SMB-RES4, SMB-RES8, and SMB-PS cells in comparison to SMB-S15 cells, were selected. The expression levels of those miRNAs in the exosomes of SMB-PS, SMB-RES4, SMB-RES8, and SMB-S15 cells were evaluated using individual specific qRT-PCR assays, with mmu-sno-135 as an internal control [13]. The mean level of each miRNA in each cell type was averaged based on four replicates after normalizing to that of the internal control.

**Fig. 4:**
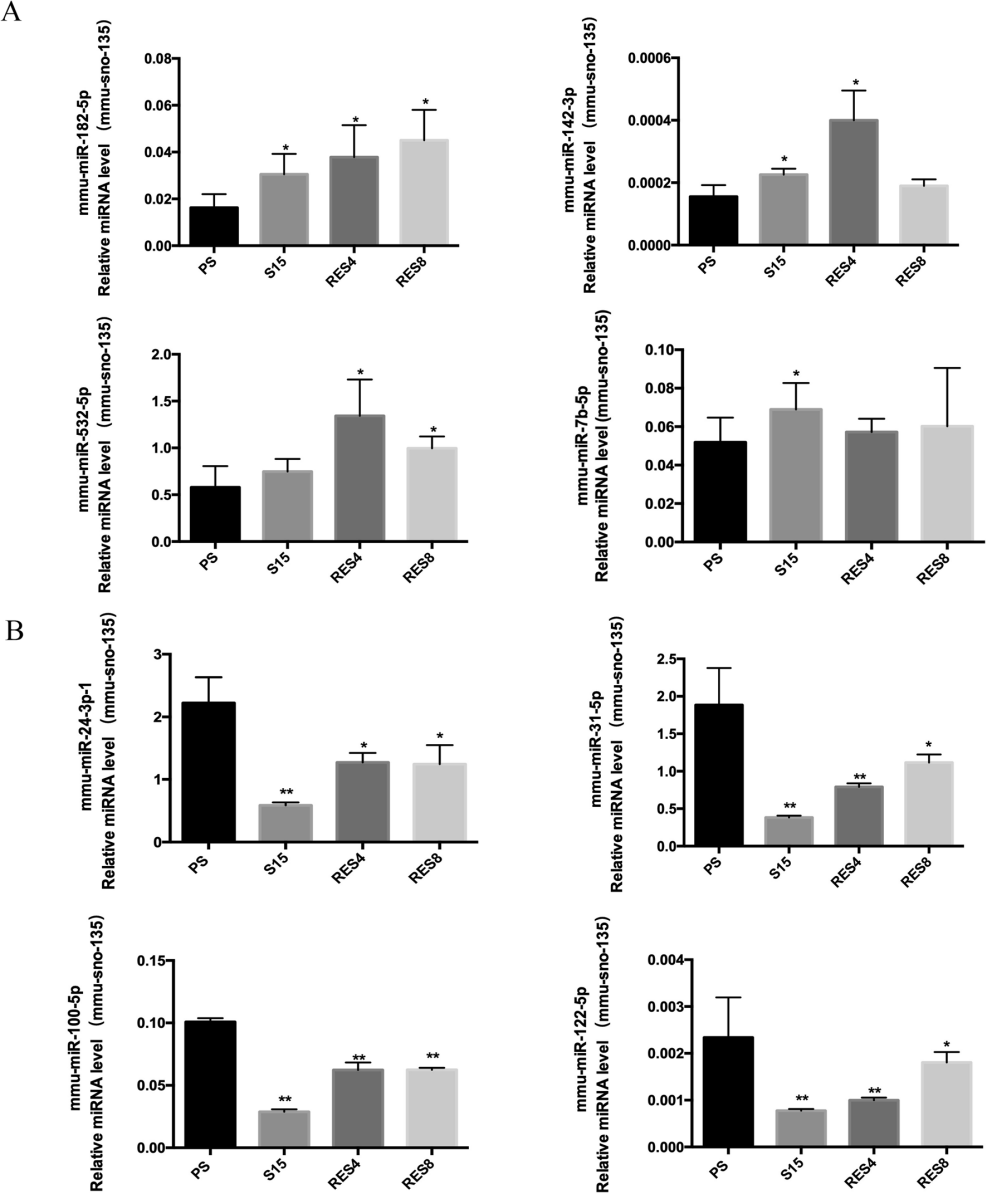
Confirmation of differential expression of eight selected miRNAs by qRT-PCR. (A) Four miRNAs that were upregulated in SMB-PS, -RES4, -RES8, and -S15 cells. (B) Four miRNAs that were downregulated in SMB-PS, -RES4, -RES8, and -S15 cells. The expression levels of those eight miRNAs in the exosomes of SMB-PS, -RES4, -RES8, and -S15 cells were evaluated using individual specific qRT-PCR assays. The *y*-axis shows relative miRNA level compared to the internal control mmu-sno-135. The data for each miRNA in each tested group are the average for three independent preparations. Each test was repeated three times. Data are presented as the mean and SD. *, *P* < 0.05; **, *P* < 0.01

For the four upregulated miRNAs selected from the deep-sequencing data, the expression levels measured by qRT-PCR in the exosomes of SMB-RES4, SMB-RES8, and SMB-PS cells were also higher than those in SMB-PS cells. Statistical analysis revealed significant differences in mmu-miR-182-5p levels in all three cell types, mmu-miR-142-3p in SMB-RES4 and SMB-RES8 cells, mmu-miR-532-5p in SMB-RES4 and SMB-RES8 cells, and mmu-miR-7b-5p in SMB-PS cells (Fig. 4A). All four downregulated miRNAs selected from the Solexa sequencing data showed significantly decreased expression in the exosomes of SMB-RES4, SMB-RES8, and SMB-PS cells compared with that of SMB-PS cells (Fig. 4B). This suggests that measurement of miRNA expression in exosomes by Solexa sequencing is reliable.

### Potential involvement of significant pathways in the exosomes of SMB-PS, SMB-RES4, and SMB-RES8 cells

To identify biological pathways that are possibly affected by the differentially expressed miRNAs, Kyoto Encyclopedia of Genes and Genomes (KEGG) pathway analysis was conducted. Hundreds of biological pathways were found to be potentially involved. Among these, 86 pathways in SMB-PS cells, 103 in SMB-RES4 cells, and 104 in SMB-RES8 cells were significantly affected (*P* < 0.05). Table 2 summarizes the top 15 KEEG pathways affected by miRNAs with at least a 4-fold change in SMB-PS, SMB-RES4, and SMB-RES8 cells in comparison to SMB-S15 cells. Nine of these pathways were affected in all three cell types, fatty acid metabolism, prion diseases, protein processing in the endoplasmic reticulum, proteoglycans in cancer, the adherens junction, the thyroid hormone signaling pathway, renal cell carcinoma, fatty acid degradation, and the FoxO signaling pathway.

**Table 2:** KEGG pathways associated with miRNAs with a 4-fold change in the cell lines SMB-PS, SMB-RES8, and SMB-RES4 compared with SMB-S15

	KEGG pathway	*P*-value	No. of genes	No. of miRNAs	Changed in
PS/S15					
1	Fatty acid metabolism	2.06E-18	33	53	All
2	Biosynthesis of unsaturated fatty acids	4.11E-14	20	52	PS & RES4
3	Prion diseases	1.94E-09	26	45	All
4	Protein processing in endoplasmic reticulum	1.94E-09	126	59	All
5	Proteoglycans in cancer	3.15E-09	139	63	All
6	Adherens junction	1.39E-07	58	56	All
7	Hepatitis B	8.56E-07	93	59	One
8	Hippo signaling pathway	1.19E-06	103	61	PS & RES8
9	Thyroid hormone signaling pathway	4.44E-06	82	58	All
10	Renal cell carcinoma	4.47E-06	51	55	All
11	Cell cycle	8.64E-06	86	51	One
12	Pathways in cancer	8.64E-06	245	67	One
13	Chagas disease (American trypanosomiasis)	1.17E-05	72	54	One
14	Fatty acid degradation	1.43E-05	27	39	All
15	FoxO signaling pathway	1.47E-05	94	58	All
RES8/S15					
1	Fatty acid metabolism	1.39E-23	36	46	All
2	Proteoglycans in cancer	2.75E-09	147	59	All
3	Prion diseases	7.28E-08	25	42	All
4	Protein processing in endoplasmic reticulum	8.14E-08	125	61	All
5	Fatty acid degradation	2.38E-07	33	37	All
6	Adherens junction	2.65E-06	57	50	All
7	Hippo signaling pathway	2.65E-06	104	57	PS & RES8
8	Thyroid hormone signaling pathway	2.65E-06	85	57	All
9	Fatty acid biosynthesis	6.26E-06	8	19	RES8 & RES4
10	Renal cell carcinoma	6.26E-06	51	50	All
11	Endocytosis	6.26E-06	153	53	RES8 & RES4
12	Central carbon metabolism in cancer	7.98E-06	48	50	RES8 & RES4
13	FoxO signaling pathway	7.98E-06	97	53	All
14	Axon guidance	1.41E-05	95	47	One
15	Citrate cycle (TCA cycle)	1.51E-05	28	40	One
RES4/S15					
1	Fatty acid metabolism	1.54E-26	35	33	All
2	MicroRNAs in cancer	1.58E-14	107	54	One
3	Proteoglycans in cancer	1.30E-10	139	46	All
4	Prion diseases	1.71E-10	26	33	All
5	Fatty acid degradation	4.23E-09	31	29	All
6	Protein processing in endoplasmic reticulum	1.80E-07	120	45	All
7	Endocytosis	2.12E-07	146	42	RES8 & RES4
8	Biosynthesis of unsaturated fatty acids	1.74E-06	17	30	PS & RES4
9	Fatty acid biosynthesis	2.63E-06	7	17	RES8 & RES4
10	Adherens junction	4.92E-06	56	37	All
11	FoxO signaling pathway	7.27E-06	92	41	All
12	Thyroid hormone signaling pathway	7.27E-06	81	43	All
13	Primary bile acid biosynthesis	9.48E-06	13	15	One
14	Central carbon metabolism in cancer	1.16E-05	44	40	RES8 & RES4
15	Renal cell carcinoma	3.58E-05	49	42	All

To further explore the situation in the context of the prion pathway, 40 miRNAs that were differentially expressed (≥4-fold) in SMB-PS cells, including 31 that increased and nine that decreased, were selected, and their expression status in SMB-RES4 and SMB-RES8 cells was evaluated. Among the 31 increased miRNAs, 22 were also upregulated in SMB-RES8 cells, eight were unchanged (<2-fold), and only one showed a decrease, whereas nine were elevated in SMB-RES4 cells, 16 were unchanged, and six were decreased (Table 3). Of the nine downregulated miRNAs, six were in SMB-RES8 cells, while three were in SMB-RES4 cells (Table 3). These data again point out the similarities between SMB-PS and SMB-RES8 cells regarding miRNA expression.

**Table 3:** Comparison of the states of the differentially expressed miRNAs with a 4-fold change involved in the prion pathway in SMB-PS cells with those in SMB-RES8 and SMB-RES4 cells

	PS/S15			Res8/S15			Res4/S15		
	log2	*P*-value	State	log2	*P*-value	State	log2	*P*-value	State
mmu-miR-125a-3p	10.21674586	6.63E-08	↑	9.204571144	0.001123276	↑	6.64385619	0.24325	↑
mmu-miR-671-5p	5.398743692	4.58E-106	↑	4.692324899	2.15E-45	↑	-3.263034406	0.00695748	↓
mmu-miR-210-3p	5.217638891	2.94E-37	↑	3.956643631	1.23E-10	↑	0	0.977654	-
mmu-miR-135b-5p	4.422903174	0	↑	1.973303262	2.07E-72	↑	-2.120629705	1.60E-32	↓
mmu-let-7e-5p	3.694225561	0	↑	0.639116075	7.63E-94	-	-2.790198599	0	↓
mmu-miR-22-5p	3.556203508	6.69E-19	↑	3.670935724	1.36E-15	↑	1.037474705	0.0981554	↑
mmu-miR-362-5p	3.555816155	1.55E-47	↑	-1.089267338	0.1888184		-0.454031631	0.351976	-
mmu-miR-148b-3p	3.464207441	3.44E-140	↑	0.448627861	0.1162552	-	1.544946822	2.62E-16	↑
mmu-miR-31-5p	3.459851363	0	↑	2.563749024	0	↑	-1.526884864	8.90E-170	↓
mmu-miR-17-5p	3.308922385	0	↑	1.648061472	2.31E-35	↑	1.269186633	2.14E-29	↑
mmu-miR-194-5p	3.162001962	0	↑	4.383943432	0	↑	2.127862863	3.26E-155	↑
mmu-miR-23b-3p	3.05094824	0	↑	1.808920285	3.48E-247	↑	0.843004877	6.50E-62	-
mmu-miR-146a-5p	3.032682842	0	↑	3.737874604	0	↑	-1.407991694	1.32E-230	↓
mmu-miR-30d-5p	3.016116391	0	↑	2.113930276	0	↑	0.226109752	1.42E-08	-
mmu-miR-30b-5p	2.940106944	0	↑	2.724670231	0	↑	-0.059808907	0.0981782	↓
mmu-miR-101a-3p	2.928916902	2.81E-14	↑	0.674599713	0.300514	-	1.106915204	0.0359468	↑
mmu-miR-146b-5p	2.882208449	0	↑	-4.12404848	1.57E-59	↓	0.57756305	1.24E-12	-
mmu-miR-218-5p	2.869954548	0	↑	3.464254182	0	↑	-1.405708043	5.13E-109	↓
mmu-miR-34c-5p	2.841858043	0	↑	1.554047352	7.96E-55	↑	-2.101409518	3.41E-45	↓
mmu-miR-24-3p	2.759867817	0	↑	1.001540573	0	↑	-0.953969401	0	-
mmu-miR-20a-5p	2.741910391	0	↑	1.051515696	3.69E-20	↑	1.357937738	1.32E-57	↑
mmu-miR-148a-3p	2.718486174	2.63E-23	↑	-2.196397213	0.01929808	↓	3.42076411	1.16E-48	↑
mmu-miR-221-3p	2.69768824	0	↑	3.175754213	0	↑	0.273822794	7.30E-06	-
mmu-let-7f-5p	2.471652907	0	↑	0.541071715	1.01E-123	-	-1.203424917	0	↓
mmu-miR-152-3p	2.403095197	1.19E-103	↑	2.356462338	2.17E-68	↑	-1.531593851	9.87E-13	↓
mmu-miR-30c-5p	2.276974859	0	↑	2.155307905	0	↑	-0.143892461	0.01554142	-
mmu-miR-421-3p	2.242502255	8.46E-286	↑	1.85363826	2.62E-116	↑	-0.910252606	2.81E-18	-
mmu-miR-26a-5p	2.174155078	0	↑	1.61359508	0	↑	-0.326553556	0	-
mmu-miR-106b-5p	2.158591911	1.29E-08	↑	0.872620694	0.1190816	-	-0.062284278	0.895058	-
mmu-miR-93-5p	2.110890703	0	↑	3.071621478	0	↑	0.209492027	3.67E-15	-
mmu-miR-19a-3p	2.1020645	4.46E-14	↑	-0.327361981	0.626248	-	1.631218092	2.82E-08	↑
mmu-miR-532-3p	-2.107148858	6.83E-42	↓	-6.306821202	2.03E-55	↓	-6.984893108	3.82E-113	↓
mmu-miR-185-5p	-2.308842336	1.09E-157	↓	2.059393123	0	↑	0.988783014	1.10E-100	-
mmu-miR-125b-5p	-2.512270351	0	↓	4.572591519	0	↑	-2.079360589	0	↓
mmu-miR-133a-3p	-2.553801569	1.07E-62	↓	-2.693526333	5.22E-39	↓	0.96201955	5.05E-34	-
mmu-let-7a-5p	-2.728511475	0	↓	0.33161104	3.52E-27	-	-0.403900405	2.55E-51	-
mmu-miR-150-5p	-3.783567262	0	↓	-2.940713659	5.88E-209	↓	3.652781214	0	↑
mmu-miR-29c-3p	-4.252387162	5.95E-15	↓	-0.688798312	0.0789432	↓	2.69632361	9.80E-68	↑
mmu-miR-532-5p	-5.383928243	0	↓	-2.366335049	0	↓	-2.712640255	0	↓
mmu-miR-1a-3p	-11.63934071	1.83E-18	↓	-11.63934071	2.00E-10	↓	0.369087913	0.1167654	-

↑, increased; ↓, decreased; -, changed less than 2-fold

### Changes in expression of miRNAs and the target genes in the KEGG pathway of prion disease in the exosomes of SMB-RES8 and SMB-PS cells

Differentially expressed miRNAs (≥4-fold) affecting the KEGG pathway “prion disease” in SMB-RES8 and SMB-PS cells were selected, and their potentially targeted genes were identified using the software DIANA-miRPath v3.0 (http://snf-515788.vm.okeanos.grnet.gr/). In total, 45 and 42 differentially expressed miRNAs were identified in the exosomes of the cell lines SMB-PS and SMB-RES8 that are potentially involved in the regulation of the transcription of 26 and 25 genes, respectively. Of these genes, 23 were identified in both cell lines. The possible transcription status of these genes in those two cell lines was analyzed. As shown in Table 4, 10 of these genes showed similar transcription activity in SMB-PS and SMB-RES8 cells, although the numbers and/or the types of miRNAs participating in transcriptional regulation varied for some genes. Those genes included *Map2K1, C6, C8b, IL6, Prkacb, Stip1*, and *Ncam1*, whose transcription was possibly downregulated, and *Mapk3, Elk1*, and *Bax*, whose transcription was upregulated. Analysis of the status of the differentially expressed miRNAs involved in the regulation of the remaining 12 genes also predicted similar regulating activity, despite a small portion of them showing the opposite effect (Table 4). These included *Notch1, Hc, C9, C8a, Prkx, IL1a, Prkaca, Hspa5, Egr1, Lamc1*, and *Prnp*, whose transcription was possibly decreased, and *Sod1*, whose transcription was possibly increased.

**Table 4:** The differentially expressed miRNAs (≥4-fold) and the possible transcriptional status of the target genes in the context of the prion pathway

	RES8/S15				PS/S15			
Gene	miRNA	log2 (RES8/S15)	*P*-value	Status	miRNAs	log2 (PS/S15)	*P*-value	Status
*Bax*	mmu-miR-532-5p	-6.306821202	2.03E-55	-	mmu-miR-532-5p	-5.383928243	0	-
*C1qb*	\	\	\		mmu-let-7e-5p	3.694225561	0	+
*C6*	mmu-miR-146a-5p	3.737874604	0	+	mmu-miR-146a-5p	3.032682842	0	+
	mmu-miR-152-3p	2.356462338	2.17E-68	+	mmu-miR-148a-3p	2.718486174	2.63E-23	+
	mmu-miR-27a-3p	3.713500895	0	+	mmu-miR-148b-3p	3.464207441	3.44E-140	+
	mmu-miR-27b-3p	3.272199672	0	+	mmu-miR-152-3p	2.403095197	1.19E-103	+
	mmu-miR-30a-5p	2.238911473	0	+	mmu-miR-30d-5p	3.016116391	0	+
	mmu-miR-671-5p	4.692324899	2.15E-45	+	mmu-miR-671-5p	5.398743692	4.58E-106	+
	mmu-miR-92a-3p	2.887224857	0	+				
*C8a*	mmu-miR-152-3p	2.356462338	2.17E-68	+	mmu-miR-107-3p	6.781359714	0.1910188	+
	mmu-miR-193b-3p	2.423957878	7.10E-53	+	mmu-miR-152-3p	2.403095197	1.19E-103	+
	mmu-miR-194-5p	4.383943432	0	+	mmu-miR-185-5p	-2.308842336	1.09E-157	-
	mmu-miR-214-3	2.333741753	2.22E-158	+	mmu-miR-194-5p	3.162001962	0	+
	mmu-miR-27a-3p	3.713500895	0	+				
	mmu-miR-27b-3p	3.272199672	0	+				
	mmu-miR-3473d	3.025816292	5.32E-19	+				
	mmu-miR-350-5p|	2.087462841	6.29E-08	+				
*C8b*	mmu-miR-152-3p	2.356462338	2.17E-68	+	mmu-miR-148b-3p	3.464207441	3.44E-140	+
					mmu-miR-152-3p	2.403095197	1.19E-103	+
*C9*	mmu-miR-27a-3p	1.487307786	3.98E-07	+	mmu-miR-223-3p	-1.683478241	7.36E-16	-
	mmu-miR-27b-3p	3.272199672	0	+	mmu-miR-421-3p	2.242502255	8.46E-286	+
	mmu-miR-671-5p	4.692324899	2.15E-45	+	mmu-miR-671-5p	5.398743692	4.58E-106	+
*Egr1*	mmu-let-7i-5p	1.608896651	0	+	mmu-let-7a-5p	-2.728511475	0	-
	mmu-miR-124-3p	2.807354922	0.05243	+	mmu-let-7e-5p	3.694225561	0	+
	mmu-miR-181a-5p	7.226686188	0	+	mmu-let-7f-5p	2.471652907	0	+
	mmu-miR-181c-5p|	2.718246106	0	+	mmu-miR-125a-3p	10.21674586	6.63E-08	+
	mmu-miR-191-5p	2.341681636	0	+	mmu-miR-17-5p	3.308922385	0	+
	mmu-miR-204-5p	3.286107619	2.07E-94	+	mmu-miR-185-5p	-2.308842336	1.09E-157	-
	mmu-miR-23a-3p	2.926253102	0	+	mmu-miR-20a-5p	2.741910391	0	+
	mmu-miR-27a-3p	3.713500895	0	+	mmu-miR-23b-3p	3.05094824	0	+
	mmu-miR-27b-3p	3.272199672	0	+	mmu-miR-26a-5p	2.174155078	0	+
	mmu-miR-93-5p	3.071621478	0	+	mmu-miR-362-5p	3.555816155	1.55E-47	+
	mmu-miR-9-5p	3.759333407	7.04E-21	+	mmu-miR-93-5p	2.110890703	0	+
*Elk1*	mmu-miR-1a-3p	-11.63934071	2.00E-10	-	mmu-miR-1a-3p	-11.63934071	1.83E-18	-
	mmu-miR-532-3p	-6.306821202	2.03E-55	-	mmu-miR-532-3p	-2.107148858	6.83E-42	-
*Fyn*	mmu-miR-138-5	3.550455792	0	+	mmu-miR-101a-3p	2.928916902	2.81E-14	+
	mmu-miR-27a-3p	3.713500895	0	+	mmu-miR-125a-3p	10.21674586	6.63E-08	+
	mmu-miR-27b-3p	3.272199672	0	+	mmu-miR-17-5p	3.308922385	0	+
	mmu-miR-30a-5p	2.238911473	0	+	mmu-miR-19a-3p	2.1020645	4.46E-14	+
	mmu-miR-30b-5p	2.724670231	0	+	mmu-miR-20a-5p|	2.741910391	0	+
	mmu-miR-9-5p	3.759333407	7.04E-21	+	mmu-miR-30b-5p|	2.940106944	0	+
					mmu-miR-30c-5p	2.276974859	0	+
					mmu-miR-30d-5p	3.016116391	0	+
*Hc*	mmu-miR-27a-3p	1.487307786	3.98E-07	+	mmu-let-7e-5p	3.694225561	0	+
	mmu-miR-27b-3p	3.272199672	0	+	mmu-miR-185-5p	-2.308842336	1.09E-157	-
					mmu-miR-22-5p	3.556203508	6.69E-19	+
					mmu-miR-26a-5p	2.174155078	0	+
*Hspa5*	mmu-miR-146a-5p	3.737874604	0	+	mmu-miR-107-3p	6.781359714	0.1910188	+
	mmu-miR-146b-5p	-4.12404848	1.57E-59	-	mmu-miR-146a-5p	3.032682842	0	+
	mmu-miR-150-5p	-2.940713659	5.88E-209	-	mmu-miR-146b-5p	2.882208449	0	+
	mmu-miR-181a-5p	2.718246106	0	+	mmu-miR-150-5p	-3.783567262	0	-
	mmu-miR-181c-5p|	2.341681636	0	+	mmu-miR-181d-5p	2.294929227	0	+
	mmu-miR-301a-3p	3.179452228	0	+	mmu-miR-22-5p	3.556203508	6.69E-19	+
	mmu-miR-30a-5p	2.238911473	0	+	mmu-miR-26a-5p	2.174155078	0	+
	mmu-miR-30b-5p	2.724670231	0	+	mmu-miR-30b-5p	2.940106944	0	+
	mmu-miR-345-5p|	2.472764773	4.95E-234	+	mmu-miR-30c-5p	2.276974859	0	+
					mmu-miR-30d-5p	3.016116391	0	+
*Il1a*	mmu-let-7i-5p	1.608896651	0	+	mmu-let-7a-5p	-2.728511475	0	-
	mmu-let-7g-5p	2.807354922	0.05243	+	mmu-let-7f-5p	2.471652907	0	+
*Il6*	mmu-miR-181a-5p	2.718246106	0	+	mmu-miR-26a-5p	2.174155078	0	+
*Lamc1*	mmu-miR-124-3p	7.226686188	0	+	mmu-miR-106b-5p	2.158591911	1.29E-08	+
	mmu-miR-153-3p	5.459431619	6.70E-51	+	mmu-miR-17-5p	3.308922385	0	+
	mmu-miR-221-3p	3.175754213	0	+	mmu-miR-20a-5p	2.741910391	0	+
	mmu-miR-27a-3p	3.713500895	0	+	mmu-miR-210-3p	5.217638891	2.94E-37	+
	mmu-miR-27b-3p	3.272199672	0	+	mmu-miR-221-3p	2.69768824	0	+
	mmu-miR-30a-5p	2.238911473	0	+	mmu-miR-29c-3p	-4.252387162	5.95E-15	-
	mmu-miR-93-5p	3.071621478	0	+	mmu-miR-31-5p	3.459851363	0	+
					mmu-miR-93-5p	2.110890703	0	+
*Map2k1*	mmu-miR-152-3p	2.356462338	2.17E-68	+	mmu-miR-101a-3p	2.928916902	2.81E-14	+
	mmu-miR-181a-5p	2.718246106	0	+				
	mmu-miR-301a-3p	3.179452228	0	+				
	mmu-miR-345-5p	2.472764773	4.95E-234	+				
*Map2k2*	mmu-miR-181a-5p	7.226686188	0	+	mmu-miR-152-3p	2.403095197	1.19E-103	+
	mmu-miR-152-3p	2.356462338	2.17E-68	+				
*Mapk3*	mmu-miR-1a-3p	-11.63934071	2.00E-10	-	mmu-miR-1a-3p	-11.63934071	1.83E-18	-
*Ncam1*	mmu-let-7i-5p	1.608896651	0	+	\	\	\	
*Ncam2*	mmu-let-7i-5p	1.608896651	0	+	mmu-miR-125a-3p	10.21674586	6.63E-08	+
					mmu-miR-17-5p	3.308922385	0	+
					mmu-miR-19a-3p	2.1020645	4.46E-14	+
					mmu-miR-30c-5p	2.276974859	0	+
					mmu-miR-30d-5p	3.016116391	0	+
*Notch1*	mmu-miR-143-3p	2.909192258	0	+	mmu-miR-107-3p	6.781359714	0.1910188	+
	mmu-miR-146b-5p	-4.12404848	1.57E-59	-	mmu-miR-125a-3p	10.21674586	6.63E-08	+
	mmu-miR-181a-5p	2.718246106	0	+	mmu-miR-146b-5p	2.882208449	0	+
	mmu-miR-1a-3p	-11.63934071	2.00E-10	-	mmu-miR-1a-3p	-11.63934071	1.83E-18	-
	mmu-miR-214-3	2.333741753	2.22E-158	+	mmu-miR-218-5p	2.869954548	0	+
	mmu-miR-218-5p	3.464254182	0	+	mmu-miR-24-3p	2.759867817	0	+
	mmu-miR-24-3p	1.001540573	0	+	mmu-miR-26a-5p	2.174155078	0	+
	mmu-miR-30a-5p	2.238911473	0	+	mmu-miR-30b-5p	2.940106944	0	+
	mmu-miR-30b-5p	2.724670231	0	+	mmu-miR-30c-5p	2.276974859	0	+
	mmu-miR-500-3p	2.619608644	1.65E-07	+	mmu-miR-30d-5p	3.016116391	0	+
	mmu-miR-92a-3p	2.887224857	0	+	mmu-miR-34c-5p	2.841858043	0	+
					mmu-miR-362-5p	3.555816155	1.55E-47	+
*Prkaca*	mmu-miR-154-5p	3.993695423	5.41E-23	+	mmu-miR-17-5p	3.308922385	0	+
	mmu-miR-1a-3p	-11.63934071	2.00E-10	-	mmu-miR-1a-3p	-11.63934071	1.83E-18	-
	mmu-miR-204-5p	3.286107619	2.07E-94	+	mmu-miR-22-5p	3.556203508	6.69E-19	+
	mmu-miR-214-3	2.333741753	2.22E-158	+				
	mmu-miR-23a-3p	2.926253102	0	+				
*Prkacb*	mmu-miR-146a-5p	3.737874604	0	+	mmu-miR-146a-5p	3.032682842	0	+
					mmu-miR-19a-3p	2.1020645	4.46E-14	+
					mmu-miR-23b-3p	3.05094824	0	+
					mmu-miR-26a-5p	2.174155078	0	+
					mmu-miR-362-5p	3.555816155	1.55E-47	+
*Prkx*	mmu-miR-124-3p	7.226686188	0	+	mmu-miR-101a-3p	2.928916902	2.81E-14	+
	mmu-miR-133a-3p	-2.693526333	5.22E-39	-	mmu-miR-125a-3p	10.21674586	6.63E-08	+
	mmu-miR-204-5p	3.286107619	2.07E-94	+	mmu-miR-133a-3p	-2.553801569	1.07E-62	-
	mmu-miR-218-5p	3.464254182	0	+	mmu-miR-17-5p	3.308922385	0	+
	mmu-miR-222-3p	1.144982994	8.32E-58	+	mmu-miR-20a-5p|	2.741910391	0	+
	mmu-miR-92a-3p	2.887224857	0	+	mmu-miR-218-5p	2.869954548	0	+
	mmu-miR-9-5p	3.759333407	7.04E-21	+	mmu-miR-31-5p	3.459851363	0	+
*Prnp*	mmu-let-7i-5p	1.608896651	0	+	mmu-miR-152-3p	2.403095197	1.19E-103	+
	mmu-miR-152-3p	2.356462338	2.17E-68	+	mmu-miR-26a-5p	2.174155078	0	+
	mmu-miR-181a-5p	2.718246106	0	+	mmu-miR-532-3p	-2.107148858	6.83E-42	-
	mmu-miR-193b-3p	2.423957878	7.10E-53	+				
	mmu-miR-27a-3p	3.713500895	0	+				
	mmu-miR-27b-3p	3.272199672	0	+				
	mmu-miR-301a-3p	3.179452228	0	+				
	mmu-miR-532-3p	-6.306821202	2.03E-55	-				
	mmu-miR-9-5p	3.759333407	7.04E-21	+				
*Sod1*	mmu-miR-1a-3p	-11.63934071	2.00E-10	-	mmu-miR-125a-3p	10.21674586	6.63E-08	+
					mmu-miR-1a-3p	-11.63934071	1.83E-18	-
*Stip1*	mmu-miR-23a-3p	2.926253102	0	+	mmu-miR-125a-3p	10.21674586	6.63E-08	+
	mmu-miR-27a-3p	3.713500895	0	+	mmu-miR-135b-5p	2.516944077	1.90E-13	+
	mmu-miR-27b-3p	3.272199672	0	+	mmu-miR-23b-3p	3.05094824	0	+

## Discussion

Using deep-sequencing techniques, we have comparatively analyzed the miRNA expression profiles in exosomes extracted from four SMB cell lines, including SMB-S15 cells, in which prions persistently replicate, SMB-PS cells, which lack prions due to treatment with **??**pentosan polysulfate**??**, SMB-RES4 cells, which are SMB-S15 cells that were exposed to resveratrol for 4 days and express a low amount of PrP^Sc^, and SMB-RES8 cells, which were exposed to resveratrol for 8 days and do not have detectable PrP^Sc^. Routine qRT-PCR tests of selected differentially expressed miRNAs (four increased and four decreased) in deep-sequencing assays showed similar expression patterns in all four SMB cell lines. In general, the exosomal miRNA expression profiles differed between SMB-S15 and SMB-PS cells. After treatment with resveratrol, the exosomal miRNA expression profiles of the treated cells differed from those of SMB-S15 and SMB-PS cells, with the profile in SMB-RES8 cells (long exposure time) being similar to that in SMB-PS cells. To our knowledge, this is the first description of the changes in exosomal miRNA expression profiles in cultured cells that occur together with the removal of prion agent.

Exosomes, which contain cell-specific proteins, lipids, and nucleic acids that can be transmitted as signal molecules, play an important role in transmission of information and transport of material between cells [5]. The biological and pathological significance of exosomes in the development of some neurodegenerative diseases, including **??**Alzheimer’s disease**??** (AD) and **??**Parkinson’s disease**??** (PD), as well as prion diseases, has been investigated, although mostly based on changes in the levels of proteins [14]. It has been shown that PrP protein, both the PrP^C^ and PrP^Sc^ isoforms, is often found at high levels in exosomes, despite not being a marker protein of exosomes [14, 15]. Exosomal PrP^Sc^ seems to be able to induce protein aggregation in cells, whereas exosomes containing PrP^C^ cannot [16]. KEGG pathway analysis based on exosomal miRNAs with at least a 4-fold change in this study indicated that the pathway “prion disease” is significantly involved in cells containing the prion agent. More interestingly, the process of eliminating prion replication by resveratrol treatment was accompanied by increasing levels of exosomal miRNAs, which reflects changes in the expression and probably the functional activity of proteins of the pathway “prion disease”.

It is now understood that aggregates of misfolded prion protein are not the only important factor in the disease process. Other events, such as ER stress, autophagy, neuroinflammation, mitochondrial dysfunction, oxidative stress, glutamate excitotoxicity, dysregulated Ca^2+^ homeostasis, aberrant expression of various kinases, and defective RNA processing, could also be components of a comprehensive network of events that affect the course of disease [17]. In the context of the KEGG pathway “prion disease”, changes in exosomal miRNA expression and their potential influence on target proteins, together with the removal of prions from SMB cells coincide with the abnormal observations in prion-infected brain tissues. As an important event in the process of innate immunity, increases in various complement components are observable in the brains of prion-infected experimental animals [18] and the cerebrospinal fluid in human sporadic CJD cases [19]. Significantly increased expression of miRNAs targeting different complement subunits, including C1qb, C6, C8a, C8b, and C9, in the exosomes of SMB cells in which prion replication has been eliminated reflect decreased expression of complement after the removal of prions. Heat shock protein 70 (Hsp70) selectively mediates the degradation of cytosolic forms of PrP proteins, and its expression increases in the brains of scrapie-infected rodents [20]. The number of miRNAs with increased expression that target the expression of the gene *HspA5*, which encodes heat shock protein A5, an Hsp70-type molecular chaperone of the endoplasmic reticulum (ER), indicates a relatively low level of Hsp70 activity in cells lacking prions. The genes *Map2K1*, *Map2K2*, and *Map3K* encode mitogen-activated protein kinase (MAPK) kinase 1, MAPK kinase 2, and MAPK3, respectively. A body of evidence has indicated an activation of the MAPK pathway in response to an accumulation of misfolded prion protein in cultured cells and in the brains of prion-infected animals [21]. In line with those findings, our data suggest that, during the process of removal of prions, exosomal miRNAs targeting those three genes are expressed more actively, which implies the inactivation of the MAPK pathway after eliminating prions. CAMP-dependent protein kinase is one of numerous kinases associated with tau phosphorylation. Aberrant tau phosphorylation is a hallmark of AD and is also detected in prion diseases [22, 23]. Increasing expression of exosomal miRNAs targeting the genes encoding cAMP-dependent protein kinase catalytic subunits alpha (*Prkaca*) and beta (*Prkacb*), as well as a serine threonine protein kinase that shows similarity to the catalytic subunit of cAMP-dependent protein kinases (*Prkx*) during prion elimination may help the cells to recover their normal tau phosphorylation process. On the other hand, expression of a few genes is likely to increase, as the expression of the related miRNAs in exosomes of the cells in which prions are removed decreases. For example, *Sod1*, encoding superoxide dismutase 1 (SOD1), which may release ROS stress, is frequently monitored in prion diseases [24]. Abnormally active exosomal miRNAs in prion-infected cells return to a relatively normal and silent state when prions are eradicated, at least in the context of the KEGG pathway “prion disease”.

In a published review, the differentially expressed miRNAs in the brains and/or cultured cells of prion-infected mice and humans with prion disease have been summarized [25]. Bellingham and colleagues described the changes in the levels of exosomal miRNAs in a cultured neuronal cell line infected with a mouse-adapted human Fukuoka-1 prion strain. The changes in the miRNA profile described in that study were similar to those in our study here, including increased let-7b, let-7i, miR-128a, miR-21, miR-222, miR-29b, and miR-342-3p and decreased miR146a in SMB-S15 cells. Compared with previous studies, we identified considerably more differentially expressed exosomal miRNAs. We presume that this was largely due to the progression of the deep-sequencing technique. It is also likely that the differences between prion strains and cell types may also influence the expression of miRNAs in exosomes.

Increased expression of miR-146a in the brain has been observed in some neurodegenerative disorders, including AD, multiple sclerosis (MS), pro-inflammatory neurodegeneration, and prion disease [26]. Certain single-nucleotide polymorphisms of miR146a show an association with the characteristics of certain diseases, e.g., diabetes, hepatocellular carcinoma, AD, and prion disease [27, 28]. As a general mechanism of the innate immune response and antiviral immunity, miR-146a actively regulates the expression of a set of inflammatory factors [29]. Our previous study also confirmed the enhanced expression of miR146a in the brain tissues of three murine scrapie models (scrapie agents 139A, ME7, and S15) [30]. In contrast to those observations *in vivo*, decreased intracellular and exosomal miR-146a was observed in prion-infected cultured cells [31]. Our data also indicate an increase in the exosomal miR146a level when prions are eliminated from cultured cells. It appears that the elevated miRNAs in the brain tissues of various forms of prion diseases are not directly derived from neuronal cells infected with prions. A growing amount of miR-146a expression during prion infection is believed to gather around activated microglia cells [26]. This may explain the inconsistent miR-146a status in the tissues and cultured cells during prion infection.

In summary, using a prion-infected cell model, changes in exosomal miRNA expression profiles during elimination of prion replication by treatment with resveratrol were monitored and analyzed. Many biological functions are likely to be involved, such as dysfunctions of various kinases, neuroinflammation, mitochondrial dysfunction, oxidative stress, and changes in the cell life cycle. Observing alterations in functional intracellular message transmission may help us to further understand the pathogenesis of prion biology and prion disease.

### Supplementary Information

Below is the link to the electronic supplementary material.

Supplementary file1 (DOCX 19 KB)

## Data Availability

All data generated or analysed during this study are included in this published article.

## Abbreviations

miRNA: MicroRNA
S15: SMB-S15
PS: SMB-PS
CJD: Creutzfeldt-Jakob disease
FFI: Fatal familial insomnia
GSS: Gerstmann-Sträussler-Scheinker syndrome
CWD: Chronic wasting disease
BSE: Bovine spongiform encephalopathy
PrP^C^: Cellular prion protein
PrP^Sc^: Pathological isoform of the prion protein
ESCRT: Endosomal sorting complex required for transport
ILVs: Intraluminal vesicles
MVB: Multivesicular body
RES4: Resveratrol for 4 days
RES8: Resveratrol for 8 days
DMSO: Dimethyl sulfoxide
PAGE: Polyacrylamide gel electrophoresis
PCR: Polymerase chain reaction
qRT-PCR: Quantitative real-time PCR
PK: Proteinase K
TSG101: Tumor susceptibility gene 101
ER: Endoplasmic reticulum
MAPK: Mitogen-activated protein kinase
