# Supplementary material for: Analysis of miRNA expression profiles in exosomes of SMB-S15 cells treated with resveratrol
Source: Arch Virol. 2023 Oct 8;168(11):270. doi: 10.1007/s00705-023-05884-6 (PMC10560638; doi:10.1007/s00705-023-05884-6)
Supplement: Supplementary file 1 — Supplementary file1 (DOCX 19 KB) [file 705_2023_5884_MOESM1_ESM.docx]

**Supplementary documents**

**Table** Comparison of the 8 changed miRNAs observed in SMB-PS, SMB-S15, SMB-RES4 and SMB-RES8 cells using TPM

| miRNA ID | TPM(SMB-PS) | TPM(SMB-S15) | TPM(SMB-RES4) | TPM(SMB-RES8) |
| --- | --- | --- | --- | --- |
| Up-regulated | | | | |
| miR-182-5p | 2.26 | 108.18 | 19.73 | 168.96 |
| miR-142-3p | 1.53 | 26.9 | 335.44 | 0.59 |
| miR-532-5p | 88.33 | 3688.35 | 562.66 | 715.31 |
| miR-7b-5p | 0.06 | 79.04 | 11.35 | 1.3 |
| Up-regulated | | | | |
| miR-24-3p-1 | 5705.66 | 842.37 | 434.84 | 1686.54 |
| miR-31-5p | 1237.53 | 112.47 | 39.03 | 664.97 |
| miR-100-5p | 5148.91 | 458.44 | 295.39 | 2385.54 |
| miR-122-5p | 1236.12 | 0.38 | 2.28 | 12.17 |

TPM：Transcripts per million, which means "for every 1,000,000 RNA molecules in the RNA-seq sample，was used to normalize the expression levels of small RNAs.
